# Aldosterone-producing Multiple Micronodules With Several Different *KCNJ5* Pathogenic Variants

**DOI:** 10.1210/jcemcr/luae213

**Published:** 2024-11-20

**Authors:** Yasushi Oiwa, Ko Aiga, Mitsuhiro Kometani, Takahiro Asano, Mikiya Usukura, Takashi Yoneda

**Affiliations:** Department of Health Promotion and Medicine of Future, Kanazawa University Graduate School of Medicine, Kanazawa, Ishikawa 920-8641, Japan; Department of Health Promotion and Medicine of Future, Kanazawa University Graduate School of Medicine, Kanazawa, Ishikawa 920-8641, Japan; Department of Health Promotion and Medicine of Future, Kanazawa University Graduate School of Medicine, Kanazawa, Ishikawa 920-8641, Japan; Department of Internal Medicine, Houju Memorial Hospital, Nomi, Ishikawa 923-1226, Japan; Department of Internal Medicine, Houju Memorial Hospital, Nomi, Ishikawa 923-1226, Japan; Department of Health Promotion and Medicine of Future, Kanazawa University Graduate School of Medicine, Kanazawa, Ishikawa 920-8641, Japan

**Keywords:** *KCNJ5*, aldosterone-producing micronodule, primary aldosteronism

## Abstract

Primary aldosteronism (PA) is the most common form of secondary hypertension. Recently, the genetic analysis of aldosterone-producing adenoma, a major cause of PA, has revealed several causative genes. Herein, we present a case of a 27-year-old Japanese female with PA. She was referred to our hospital with hypertension and hypokalemia (serum potassium, 2.8 mEq/L [2.8 mmol/L]). PA was diagnosed using several confirmatory tests. Computed tomography showed no apparent tumor in either adrenal gland. Adrenal vein sampling showed aldosterone overproduction in the right adrenal gland. Laparoscopic right adrenalectomy was performed, her blood pressure normalized, and the hypokalemia improved after surgery. Pathological findings revealed multiple aldosterone-producing micronodules with diameters of <5 mm. DNAs were extracted from 4 different micronodules and analyzed for *KCNJ5*. Two micronodules had a T158A pathogenic variant, 1 had a G151R pathogenic variant, and 1 had no pathogenic variant in the *KCNJ5* gene. In summary, in our case, multiple nodules were present in 1 adrenal gland, and genetic heterogeneity was identified. No recurrence on the left side has been observed over 17 years following the surgery.

## Introduction

Primary aldosteronism (PA) is a collection of endocrine disorders characterized by high aldosterone concentrations relative to sodium status. PA is a predominant cause of secondary hypertension [1]. Patients with PA have a higher risk of cardiovascular disorders, such as stroke, coronary artery disease, and heart failure, than those with essential hypertension [1]. The Japan Endocrine Society guideline described, in line with other international guidelines, that the prevalence of PA is greater than 5% among hypertensive patients at specialized centers [1]. Distinguishing the source of aldosterone hypersecretion is important because unilateral diseases (ie, unilateral adrenal hyperplasia and aldosterone-producing adenoma) are surgically curable, whereas bilateral diseases are treated with lifelong medications [1]. Aldosterone-producing nodules <10 mm in diameter are referred to as aldosterone-producing micronodules [2].

Over the last decade, PA has been studied genetically. Several driver genes for aldosterone-producing adenoma have been identified, including *KCNJ5*, *CACNA1D*, *ATP1A1,* and *ATP2B3* [3]. Somatic variants in *KCNJ5* are more common than variants in other genes in Asian populations [3]. In certain documented cases of PA, a single adrenal gland contained multiple micronodules, each with distinct genetic characteristics [4].

Here we present a case of a Japanese patient with PA who was treated with unilateral adrenalectomy. Notably, the adrenal lesions were not identified on computed tomography (CT). Adrenal vein sampling (AVS) was performed for lateralization. This case highlights the genetic heterogeneity of the removed gland, which contained multiple nodules, each carrying a unique pathogenic variant.

## Case Presentation

A 27-year-old Japanese woman was referred to the endocrinology department of our hospital for evaluation of persistent hypertension and hypokalemia with serum potassium levels of 2.8 mEq/L (2.8 mmol/L) (reference range, ≥3.5 mEq/L [≥3.5 mmol/L]). At the age of 25 years, she was diagnosed with hypertension at a health checkup but was left untreated. At the age of 26 years, the patient was again found to have hypertension at a local clinic, with a blood pressure reading of 158/98 mmHg. At referral, her plasma renin activity (PRA) was 0.5 ng/mL/h (reference range, 0.2-2.7 ng/mL/h), plasma aldosterone concentration (PAC) was 30 ng/dL (832 pmol/L) (reference range, 2.0-13 ng/dL [56-361 pmol/L]), and aldosterone-to-renin ratio (ARR) was 60 (reference range, <20) (Table 1). Owing to a high suspicion of PA, the patient was admitted to our hospital for further examination and treatment.

**Table 1.** Laboratory values

	At diagnosis	Reference range
Serum potassium	3.5 mEq/L (3.5 mmol/L)	3.5-5.0 mEq/L (3.5-5.0 mmol/L)
Plasma renin activity	0.3 ng/mL/h	0.2-2.7 ng/mL/h
Plasma aldosterone concentration	25 ng/dL (693 pmol/L)	2.0-13 ng/dL (56-361 pmol/L)
Aldosterone-to-renin ratio	83 [(ng/dL)/(ng/mL/h)]	<20 [(ng/dL)/(ng/mL/h)]

Aldosterone-to-renin ratio is calculated by plasma aldosterone concentration (ng/dL)/plasma renin activity (ng/mL/h).

## Diagnostic Assessment

**Figure 1.**
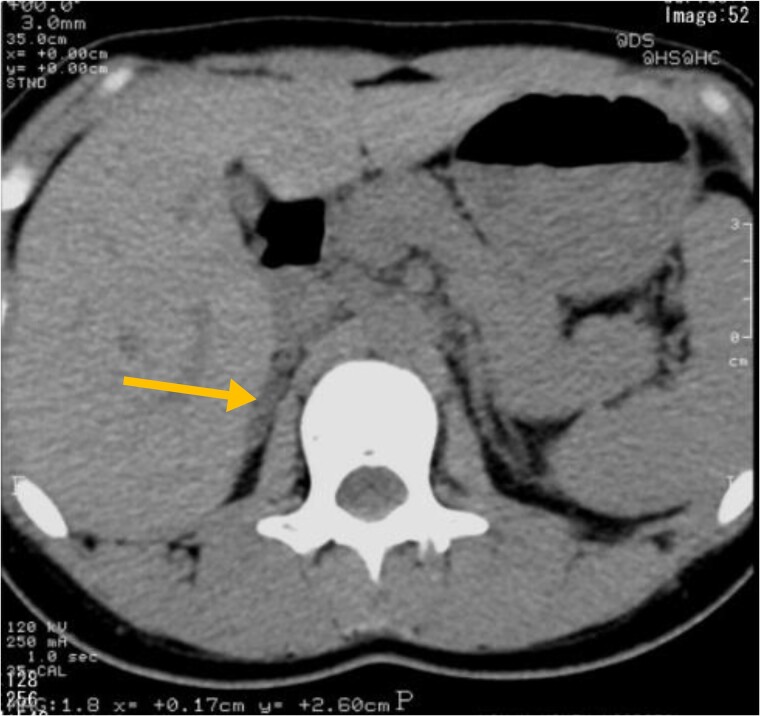
CT image of the abdomen. The arrow indicates the right adrenal gland. No abnormalities were identified on CT. Abbreviation: CT, computed tomography.

The patient's medical history was unremarkable except for hypertension. Her family history was significant for diabetes mellitus in her father. There was no known family history of hypertension. The patient was a social drinker and nonsmoker. On physical examination, the patient's height, weight, and body mass index were 156.3 cm, 51.7 kg, and 21.2 kg/m², respectively. Laboratory studies showed a relatively low serum potassium level at 3.5 mEq/L (3.5 mmol/L). Her PRA, PAC, and ARR levels were 0.3 ng/mL/h, 25 ng/dL (693 pmol/L), and 83.3, respectively (Table 1). A series of confirmatory tests for PA were performed. Captopril challenge test, furosemide upright test, and saline infusion test were positive, confirming PA in 2006 (Table 2). CT showed normal appearance of bilateral adrenal glands (Fig. 1). Following the CT scan, the patient underwent unstimulated AVS. Both veins were successfully cannulated, and AVS revealed markedly elevated aldosterone secretion from the right adrenal gland compared to the left side, suggesting that the right gland was dominant (Table 3).

**Table 2.** Confirmatory tests

Test	Procedure	Baseline	90 minutes	120 minutes	240 minutes
CCT	Administer 50 mg of captopril orally and collect blood samples in a resting supine position. Diagnose as PA if ARR is >20 [(ng/dL)/(ng/mL/h)] after 90 minutes.	PRA, 0.3 ng/mL/h PAC, 20.9 ng/dL (580 pmol/L) ARR, 70 [(ng/dL)/(ng/mL/h)]	PRA, 0.4 ng/mL/h PAC, 24.1 ng/dL (669 pmol/L) ARR, 60 [(ng/dL)/(ng/mL/h)]	—	—
FUT	Administer 40 mg of furosemide intravenously. After the injection, have the patient remain in an upright position for 2 hours, then collect blood samples. Diagnose as PA, if PRA is <2 ng/mL/h.	PRA, 0.1 ng/mL/h PAC, 21.8 ng/dL (605 pmol/L)	—	PRA, <0.1 ng/mL/h PAC, 10.1 ng/dL (280 pmol/L)	—
SIT	Administer 2 L of normal saline intravenously over 4 hours. Collect a blood sample in a recumbent position. Diagnose as PA if PAC after the load exceeds 8.5 ng/dL (236 pmol/L).	PRA, 0.3 ng/mL/h PAC, 21.8 ng/dL (605 pmol/L)	—	—	PRA, 0.3 ng/mL/h PAC, 26.0 ng/dL (721 pmol/L)

Reference range: PRA, 0.2-2.7 ng/mL/h; PAC, 2.0-13 ng/dL (56-361 pmol/L).

Abbreviations: ARR, aldosterone-to-renin ratio; CCT, captopril challenge test; FUT, furosemide upright test; PA, primary aldosteronism; PAC, plasma aldosterone concentration; PRA, plasma renin activity; SIT, saline infusion test.

**Table 3.** Unstimulated adrenal vein sampling

	Inferior vena cava	Right adrenal vein	Left adrenal vein
Aldosterone	17.3 ng/dL (480 pmol/L)	2878.3 ng/dL (79 853 pmol/L)	21.4 ng/dL (593 pmol/L)
Cortisol	13 µg/dL (359 nmol/mL)	127 µg/dL (3504 nmol/mL)	27 µg/dL (745 nmol/mL)
Aldosterone/cortisol	1.33	22.66	0.79
Selectivity index	—	9.8	2.1

Successful cannulation was confirmed by a selectivity index > 2. Selectivity index is defined as the ratio of the cortisol levels in the adrenal vein to that in the inferior vena cava. The lateralization index is used to determine the lateralization of aldosterone hypersecretion. Without ACTH stimulation, the lateralization index cutoff value is 2. To calculate the lateralization index, first, determine the aldosterone-to-cortisol ratio for each adrenal vein; then divide the higher ratio by the lower ratio to find the high side to low side ratio. Reference range: selectivity index >2.

## Treatment

The patient desired a surgical cure. Therefore, laparoscopic right adrenalectomy was performed.

## Outcome and Follow-up

**Figure 2.**
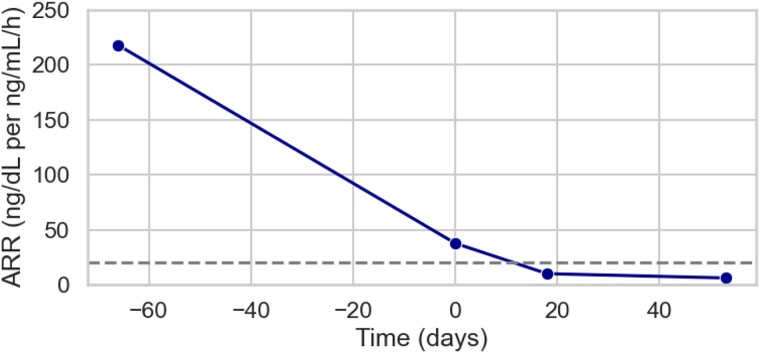
Line plots of ARR from preoperative day 66 to postoperative day 53. A horizontal dotted line indicates 20. ARR is calculated by plasma aldosterone concentration (ng/dL)/plasma renin activity (ng/mL/h). Reference range: ARR, <20 [(ng/dL)/(ng/mL/h)]. Abbreviation: ARR, aldosterone-to-renin ratio.

**Figure 3.**
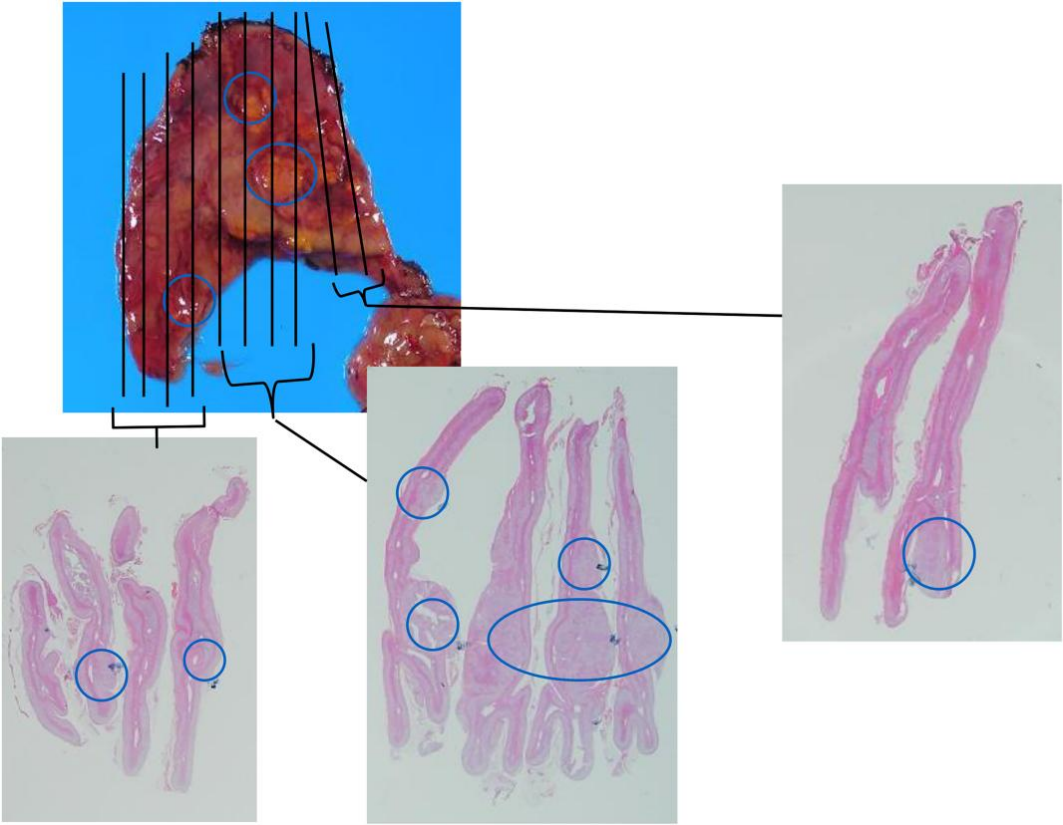
Gross and microscopic findings of the resected adrenal gland.

**Figure 4.**
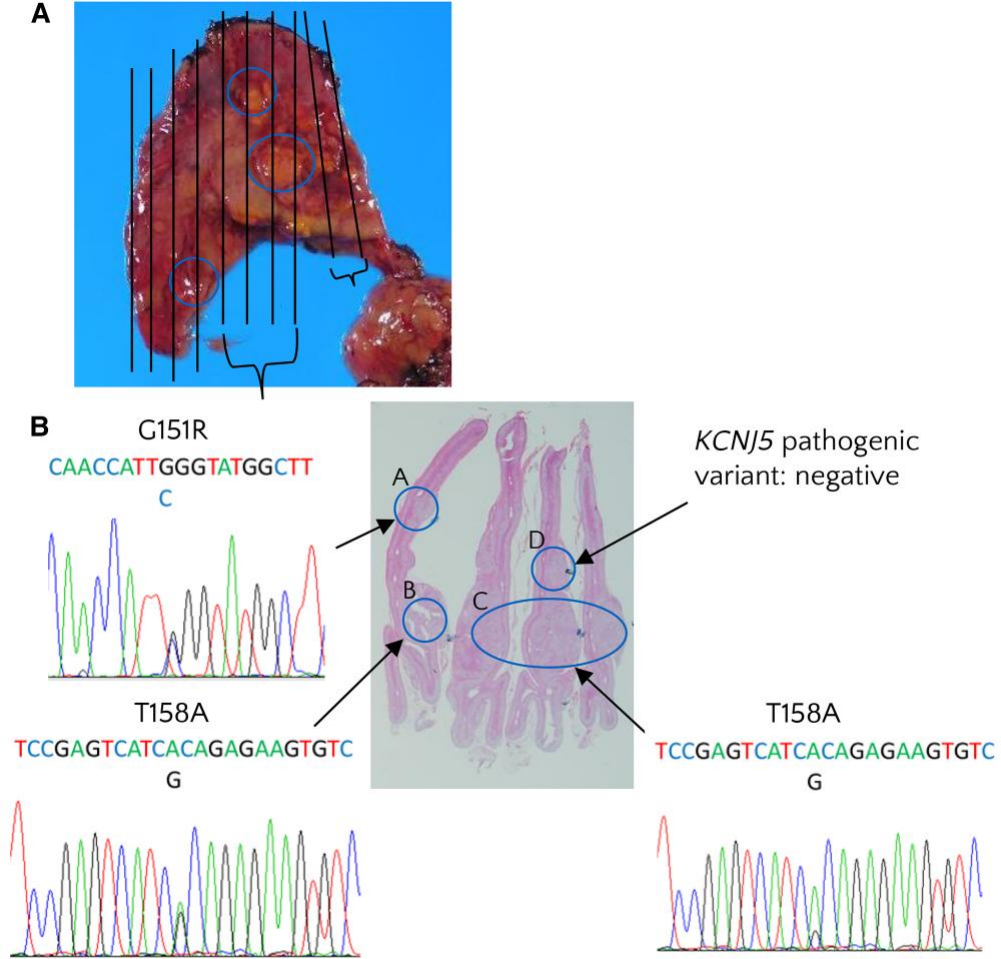
Macroscopic examination, histopathological analysis, and genotyping of nodules. (A) Macroscopic image of the right adrenal gland. The circles indicate 3 nodules identified on the gland. (B) Hematoxylin and eosin stain. Genotyping of nodules in sites A, B, C, and D revealed that sites A, B, and C harbored pathogenic variants in the *KCNJ5* gene, G151R, T158A, and T158A, respectively. Notably, site D carried no pathogenic variants in *KCNJ5*.

On postoperative day 18, the patient's PRA was 0.5 ng/mL/h, PAC was 5.0 ng/dL (140 pmol/L), and ARR was 10.1. Over the 17 years since the adrenalectomy, no recurrence of PA was documented (Fig. 2, Table 4). The adrenal gland was histopathologically analyzed, and 7 nodules were identified (Fig. 3). The largest nodule measured 5 mm in diameter. We conducted a genomic analysis of *KCNJ5* using Sanger sequencing at 4 separate sites (sites A, B, C, and D) of the gland and identified 2 pathogenic variants in the *KCNJ5* gene in 2013 (Fig. 4). G151R pathogenic variants were identified at site A. T158A pathogenic variants were found at sites B and C. Site D did not harbor any variants in *KCNJ5*. Insufficient amounts of DNA made it impossible to perform further analysis for other genes such as *CACNA1D*, *ATP1A1*, and *ATP2B3*.

**Table 4.** Postoperative aldosterone and renin status

Postoperative time (years)	PRA	PAC	ARR
0	0.4 ng/mL/h	15.1 ng/dL (419 pmol/L)	37 [(ng/dL)/(ng/mL/h)]
8	0.6 ng/mL/h	4.1 ng/dL (114 pmol/L)	6.8 [(ng/dL)/(ng/mL/h)]
13	3.0 ng/mL/h	17.9 ng/dL (497 pmol/L)	6.0 [(ng/dL)/(ng/mL/h)]
18	0.8 ng/mL/h	1.3 ng/dL (36 pmol/L)	1.6 [(ng/dL)/(ng/mL/h)]

Reference range: PRA, 0.2-2.7 ng/mL/h; PAC, 2.0-13 ng/dL (56-361 pmol/L); ARR, <20 (ng/dL)/(ng/mL/h).

Abbreviations: ARR, aldosterone-to-renin ratio; PAC, plasma aldosterone concentration; PRC, plasma renin activity.

## Discussion

Herein we describe a case of a 27-year-old woman with unilateral PA, successfully treated with unilateral adrenalectomy and followed up for 17 years. The resected adrenal gland contained multiple nodules and presented genetic heterogeneity, as revealed by DNA sequencing.

The CT scan revealed no nodules in the adrenal glands at referral. However, 7 small nodules were postoperatively identified with histopathological analysis. Therefore, the possibility of unilateral PA cannot be excluded using only CT images, which can be a pitfall for clinicians. AVS is recommended for accurate lateralization of adrenal lesions. There is robust evidence demonstrating poor diagnostic accuracy of CT in subtyping compared to AVS, with a recent study showing only a 63.8% concordance rate between the 2 modalities [5].

This case reports an exceptionally long-term follow-up after unilateral adrenalectomy, where the patient required no antihypertensive medication, maintained stable hormone levels, and showed no recurrence of PA. This favorable outcome differs from a previous report of a Chinese female harboring different pathogenic variants in *KCNJ5*; she experienced recurrence in 10 years [6]. Previous observational studies have assessed various outcomes of surgical vs medical treatment for unilateral PA, including the impact on atrial fibrillation, quality of life, and renal function [7]. While there is a growing interest in examining the long-term outcomes of adrenalectomy for unilateral PA, comprehensive studies on long-term outcomes are yet to be conducted. The lack of standardized criteria for evaluating outcomes has hindered the accumulation of evidence regarding the long-term follow-up of patients who undergo unilateral adrenalectomies [8]. Although the 2017 PASO study established a consensus on the standardized methods for evaluating clinical and biochemical outcomes, studies on outcomes spanning over a decade after adrenalectomy are rare [9].

Previous reports have described the occurrence of multiple nodules within a single gland with documented genetic heterogeneity [4, 10]. Unilateral PA has been studied histopathologically because resected tissues are available for further analysis. Over the past 10 years, advancements in DNA sequencing technology have enabled the genetic analysis of surgically collected tissues in cases of unilateral PA. In the present case, 3 nodules harboring *KCNJ5* pathogenic variants were identified (Fig. 4). Variants in *KCNJ5* are more frequent in Asian populations [3]. While the mechanisms underlying this fact are still unknown, a high salt diet among Asian populations might be associated with a higher prevalence of pathogenesis. According to a previous study, elevated salt intake led to increased aldosterone expression and salt-dependent elevated blood pressure in human aldosterone synthase-transgenic mice [11]. The presence of distinct pathogenic variants in *KCNJ5* across sites A, B, and C and the absence of pathogenic variants at site D were identified, suggesting that each nodule has a distinct and independent origin. Notably, T158A pathogenic variants, identified in site A, are rare. Previous studies elucidated the mechanisms; the T158A pathogenic variants in *KCNJ5* eliminate key hydrogen bonds that maintain the structure of the channel, leading to a decrease in K⁺ selectivity and causing membrane depolarization due to altered ion permeability [12]. Further genetic analyses of the nodules could provide clues for understanding the underlying mechanisms responsible for nodule formation.

Two theories have been proposed to explain how somatic pathogenic variants contribute to nodule formation in the adrenal cortex: the aldosterone-producing cell cluster model and the 2-hit hypothesis [3]. In the aldosterone-producing cell cluster model, it is thought that a patient's normal aldosterone-producing cells acquire pathogenic variants in the *KCNJ5* gene, leading to abnormal cell proliferation [13]. Hypothetically, in our case, this series of events occurred in at least 3 independent cells, resulting in nodule formation.

The 2-hit hypothesis may offer another explanation for the formation of the nodules in our case; her adrenal gland possibly underwent remodeling, which caused altered gene expression and/or promoted cell survival and proliferation [3]. An in vitro study suggested that such remodeling could stimulate proliferation through the activated Wnt/β-catenin signaling pathway and suppress apoptosis through the modified immune tumor microenvironment [14]. Following initial remodeling, presumably, separate micronodules acquired a unique pathogenic variant in the *KCNJ5* gene, except site D, which explains the genetic heterogeneity observed in our patient (Fig. 4). Although the heterogeneity of resected tissues has been identified, its clinical significance, such as its potential to predict poor long-term outcomes, remains uncertain. The resected adrenal glands contained clones carrying unique somatic pathogenic variants, suggesting that the left gland might have undergone some remodeling and had a higher possibility of acquiring pathogenic variants.

In summary, our findings highlight the multinodularity and genetic heterogeneity in the resected adrenal gland. Notably, CT images showed no abnormalities in her adrenal glands. This case is significant for documenting a remarkably long follow-up after unilateral adrenalectomy, during which the patient showed favorable outcomes.

## Learning Points

- Unilateral PA may still be possible even if the adrenal glands appear normal on CT, and AVS is recommended to identify the laterality of PA.
- Genetic heterogeneity may be found when multiple aldosterone-producing micronodules are present within a single adrenal gland.
- DNA sequencing of the resected glands is recommended to gain insights into the pathological mechanisms behind the formation of aldosterone-producing adenomas.
- Patients with PA who undergo unilateral adrenalectomy may show favorable outcomes even after 17 years of follow-up.

## Contributors

Y.O., K.A., and M.K. drafted the manuscript. M.K. contributed to the case detection and discussion. T.A., M.U., and T.Y. reviewed and edited the manuscript. All the authors reviewed and approved the final version of the manuscript.

## Data Availability

Restrictions apply to the availability of some or all data generated or analyzed during this study to preserve patient confidentiality or because they were used under license. The corresponding author will, on request, detail the restrictions and any conditions under which access to some data may be provided.
